# Fludarabine-induced bradycardia in a patient with refractory leukemia

**DOI:** 10.4103/0256-4947.62825

**Published:** 2010

**Authors:** Woei Chung-Lo, Ching-Yun Hsieh, Chang-Fang Chiu, Li-Yuan Bai

**To the Editor:** A 22-year-old male diagnosed with acute myelogenous leukemia in November 2005 achieved complete remission after two courses of induction chemotherapy with idarubicin (12 mg/m^2^ intravenously for 3 days) and cytosine arabinoside (200 mg/m^2^ in a 24-hour infusion for 7 days). He then received an additional two courses of cytosine arabinoside (3000 mg/m^2^) in a 3-hour continuous intravenous infusion twice daily for 3 days in April 2006 for intensification. Six months later, the leukemia relapsed and was refractory to re-induction chemotherapy (idarubicin 12 mg/m^2^ on days 1-3 and cytosine arabinoside 200 mg/m^2^ on days 1-7). With the exception of myelosuppression, no toxic events occurred during idarubicin and cytosine arabinoside treatment. Due to the refractoriness to re-induction chemotherapy, salvage chemotherapy with the FLAGI regimen (fludarabine 30 mg/m^2^ on days 1-5, cytosine arabinoside 2000 mg/m^2^ on days 1-5, and idarubicin 12 mg/m^2^ on days 7-8) was prescribed. Before chemotherapy, his vital signs were stable, with a blood pressure of 111/65 mm Hg, a respiratory rate of 19/min, a heart rate of 97/min, and a body temperature of 36.6°C. An electrocardiogram (ECG) showed a normal sinus rhythm (Figure 1a). Premedication included dexamethasone, granisetron, and metoclopramide, which were prescribed in previous chemotherapy. Thirty minutes after the fludarabine infusion had been started, he developed sudden-onset general weakness, which lessened 5 minutes later. At that time, persistent bradycardia (48 beats per minute) was noted; vital signs were otherwise normal (blood pressure, 120/70 mm Hg; respiratory rate, 20/min; and body temperature, 36.1°C). ECG revealed sinus bradycardia but no atrioventricular block, ST segment elevation or depression, or T wave inversion (Figure 1b). The patient did not have chest tightness or pain, dizziness, cold sweats, palpitations, dyspnea, or fever with chills. Investigations showed serum potassium 4.9 mmol/L (normal, 3.5-4.5 mmol/L), creatinine 1.0 gm/dL (normal, 0.5-1.3 g/dL), CPK 19 IU/L (normal, 38-174 IU/L), creatine kinase MB fraction 2.7 U/L (normal, 3-10 U/L), and troponin I < 0.04 ng/ml (normal, <0.8 ng/mL). No specific therapy was administered; however, oxygen was given at 4 L/min via nasal cannula. The persistent bradycardia resolved gradually, 3 hours after cessation of the 5-day fludarabine infusion (Figure 1c); he then received 2 days of idarubicin treatment without incident (Figure 2).

**Figure 1a:**
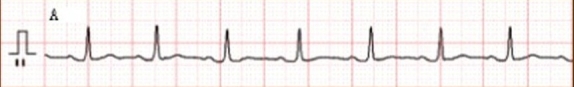
ECG findings before the start of therapy.

**Figure 1b:**
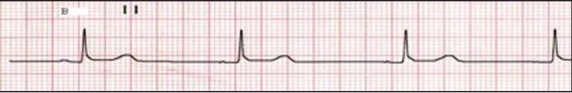
ECG on day 1-5 of fludarabine infusion.

**Figure 1c:**
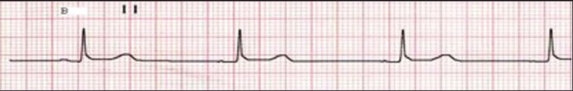
ECG on the day following infusion of fludarabine.

**Figure 2:**
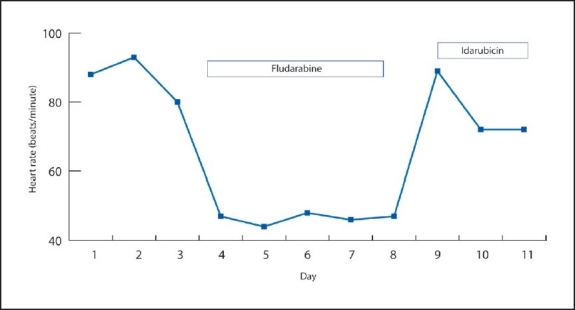
Correlation between heart rate and FLAGI regimen infusion.

Cytosine arabinoside is frequently used in the treatment of hematologic malignancies, especially acute myelogenous leukemia. Sporadic cases of pericarditis, pericardial effusion, cardiac tamponade, congestive heart failure, and sinus bradycardia have previously been reported as complications of cytosine arabinoside.^1^–^3^ Fludarabine is a purine antagonist that is commonly used for patients with non-Hodgkin lymphoma and chronic lymphocytic leukemia. The most common adverse effects are myelosuppression and immunosuppression. Cardiovascular complications of fludarabine are rare, although several cases of congestive heart failure or left ventricular failure have been reported.^4^–^5^

There are many possible etiologies for the bradycardia seen in the present patient. For example, sepsis, cardiogenic events, electrolyte imbalance, and drugs such as cytosine arabinoside may all cause bradycardia. An adverse drug reaction is considered to be the probable cause of the bradycardia in this patient because no other etiologies were apparent. Cytosine arabinoside seemed an unlikely cause of bradycardia because the patient had received cytosine arabinoside-containing chemotherapy previously. The bradycardia occurred during fludarabine treatment and subsided significantly after completion of the infusion of this drug. Other probable causes of bradycardia had already been excluded and therefore it is reasonable to speculate about a possible relationship between fludarabine and the sinus bradycardia observed in this patient. Clinicians should be aware of this potential toxic effect of fludarabine, especially in view of its increased use in the treatment of hematologic diseases.

## References

[1] Vaickus L, Letendre L (1999). Pericarditis induced by high-dose cytarabine therapy. Arch Intern Med.

[2] Conrad ME (1992). Cytarabine and cardiac failure. Am J Hematol.

[3] Stamatopoulos K, Kanellopoulou G, Vaiopoulos G, Stamatellos G, Yataganas X (1998). Evidence for sinoatrial blockade assiciated with high dose cytarabine therapy. Leuk Res.

[4] Spriano M, Clavio M, Carrara P, Canepa L, Miglino M, Pierri I (1994). Fludarabine in untreated and previously treated B-CLL patients: a report on efficacy and toxicity. Haematologica.

[5] Ritchie DS, Seymour JF, Roberts AW, Szer J, Grigg AP (2001). Acute left ventricular failure following mephalan and fludarabine conditioning. Bone Marrow Transplant.

